# Clinical perspectives of TRAIL: insights into central nervous system disorders

**DOI:** 10.1007/s00018-016-2164-7

**Published:** 2016-02-24

**Authors:** Veronica Tisato, Arianna Gonelli, Rebecca Voltan, Paola Secchiero, Giorgio Zauli

**Affiliations:** Department of Morphology, Surgery and Experimental Medicine and LTTA Centre, University of Ferrara, Via Fossato di Mortara 66, 44121 Ferrara, Italy

**Keywords:** TRAIL, Neuroinflammation, Alzheimer’s disease, Multiple sclerosis, Ischemic stroke, Therapeutic potential, Biomarker

## Abstract

The TNF-related apoptosis inducing ligand TRAIL is a member of the TNF superfamily that has been firstly studied and evaluated for its anti-cancer activity, and the insights into its biology have already led to the identification of several TRAIL-based anticancer strategies with strong clinical therapeutic potentials. Nonetheless, the TRAIL system is far more complex and it can lead to a wider range of biological effects other than the ability of inducing apoptosis in cancer cells. By virtue of the different receptors and the different signalling pathways involved, TRAIL plays indeed a role in the regulation of different processes of the innate and adaptive immune system and this feature makes it an intriguing molecule under consideration in the development/progression/treatment of several immunological disorders. In this context, central nervous system represents a peculiar anatomic site where, despite its “status” of immune-privileged site, both innate and adaptive inflammatory responses occur and are involved in several pathological conditions. A number of studies have evaluated the role of TRAIL and of TRAIL-related pathways as pro-inflammatory or protective stimuli, depending on the specific pathological condition, confirming a twofold nature of this molecule. In this light, the aim of this review is to summarize the main preclinical evidences of the potential/involvement of TRAIL molecule and TRAIL pathways for the treatment of central nervous system disorders and the key suggestions coming from their assessment in preclinical models as proof of concept for future clinical studies.

## Introduction

The acronym TRAIL designates a member of the tumor necrosis factor (TNF) family first reported in the 1990s by two independent studies. Both described TRAIL, TNF-related apoptosis-inducing ligand, as a protein able to mediate cell signals triggering caspase activation and programmed cell death in several cell types [1, 2]. Although interest in its biological features has primarily been focused on its anti-cancer activity [3–7], growing attention is also being paid to TRAIL involvement in both normal immunological homeostasis, and the development/monitoring of pathological conditions, by virtue of its involvement in different processes of the innate and adaptive immune systems. In this light, our group has extensively contributed to demonstrate an inverse correlation between the circulating levels of TRAIL and the chronic inflammation present in several pathological settings, suggesting that TRAIL may have potential as a biomarker. We have also shown that low levels of TRAIL are associated with total and cardiovascular mortality in older adults and interestingly, we have demonstrated that 17-β estradiol could play a role in regulating the amount of TRAIL in circulation by mediating downregulation of TRAIL expression [8, 9]. In addition, and in line with other reports, we have shown that TRAIL is inversely associated with outcome and mortality in patients with several pathological conditions, which include chronic kidney diseases, heart/kidney transplant, and cardiovascular disorders such as acute myocardial infarction, heart failure, and coronary heart disease [10–15]. More recently, clinical studies have shown significant changes in the circulating levels of TRAIL in patients affected by Type 1 [16, 17] and Type 2 [18, 19] diabetes mellitus [20], confirming the results of previous in vitro and in vivo preclinical studies that suggested a general protective effect of TRAIL in the development/progression of diabetes and diabetes-related complications [21–24].

However, the role of TRAIL seems to be fairly ambiguous, with different reports suggesting conflicting roles for TRAIL in the development/control of several pathological conditions. This apparently paradoxical behavior has recently been highlighted and reviewed on rheumatoid arthritis [25], for example, in which some reports implicate TRAIL in its pathogenesis, while others suggest it may have a protective effect, potentially by controlling synovial hyperplasia and immune cell hyper-activation and acting as a prognostic factor [25, 26]. A possible protective role of TRAIL has also emerged from preclinical [27] and clinical studies in atherogenesis [28], which seem to indicate that TRAIL acts to control homeostasis in atherosclerotic blood vessels. However, there may also be a harmful relationship between levels of TRAIL and vascular inflammation and atherosclerosis, and this requires further investigation [29]. Similarly, while some authors report a potential involvement of endogenous TRAIL in the development of allergic asthma in preclinical models [30], we, among others, have shown that soluble exogenous TRAIL appears to play a protective role in the resolution phase of asthma in a model of chronic allergen inhalation [31, 32].

It appears therefore that the activities and functions of TRAIL are extremely complex, being the result of the involvement of multiple pathways and of multiple levels of control, with each depending upon the specific cell type and the specific biological/pathological context. Hence, discovering the interplay between resident and immune cells may be the key to understanding the development of several diseases, and could lead to the identification of new therapeutic targets. Under normal conditions the central nervous system (CNS) has a so-called “immune privilege status” characterized by limited local inflammation. However, in presence of pathological disorders like Alzheimer’s disease, multiple sclerosis and local brain injuries such as ischemic stroke, which all share common features [33], immune responses are stimulated via activation of microglia and infiltration of circulating cells [34, 35]. In order to shed light on the involvement of TRAIL in this process, starting from our experience and knowledge of the role of TRAIL in different pathological settings, we set out to review the state of the art on the role of TRAIL in the CNS.

### TRAIL and TRAIL-mediated signalling

The TRAIL protein is encoded by a gene mapped on chromosome 3 at position 3q26. This site spans approximately 20 kb, includes five exons and four introns, and is regulated by a combination of factors, including transcription factors, epigenetics factors, and the presence of single-nucleotide polymorphisms and functionally distinct isoforms [36, 37]. Like other members of the TNF family, TRAIL protein can be detected as trans-membrane type protein and as soluble protein [38]. TRAIL exerts its biological effects through interactions with a complex ligand–receptor system of five cognate receptors, which can be bound with different affinities under normal physiological conditions [39, 40]. In humans, TRAIL is released by several cell types, and is capable of binding the transmembrane pro-apoptotic death receptors TRAIL-R1/DR4 and TRAIL-R2/DR5. It also binds to the two transmembrane decoy receptors TRAIL-R3/DcR1 and TRAIL-R4/DcR2, which lack an intact death domain. The fifth element of the TRAIL receptor system is osteoprotegerin (OPG), originally discovered as a mediator of osteoclastogenesis regulation via interaction with the receptor activator of NFκB ligand (RANKL) [41–47]. OPG is a secreted protein that lacks both transmembrane and cytoplasmic domains; it is able to bind not only RANKL, but also TRAIL, for which it acts as a soluble neutralizing receptor [3]. Interestingly, mice, unlike humans, only have one TRAIL receptor containing a death domain DD (mDR5); this, however, does show 60 % sequence homology with human DR4 and DR5 [1]. Mice also have the two decoy receptors, mDcR1 and mDcR2, that correspond to human decoy TRAIL receptors DcR1 and DcR2, albeit with differences in sequence structures. These findings suggest that TRAIL decoy receptors are a relatively recent evolutionary event [48].

As previously mentioned, the best characterized biological function of TRAIL is its ability to induce apoptosis in various cancer cells types [49–51]. This is mediated by its two death-domain-containing receptors, whereas TRAIL-DcR1, TRAIL-DcR2 and OPG are considered neutralizing or regulatory receptors [52]. Signals resulting from death-domain–ligand interaction can induce apoptosis via assembly of the death-inducing signalling complex (DISC) [53]. This is formed by the Fas-associated death domain (FADD), which promotes autocatalytic processing of caspases, activation of caspases, and thereby apoptosis [53].

**Fig. 1:**
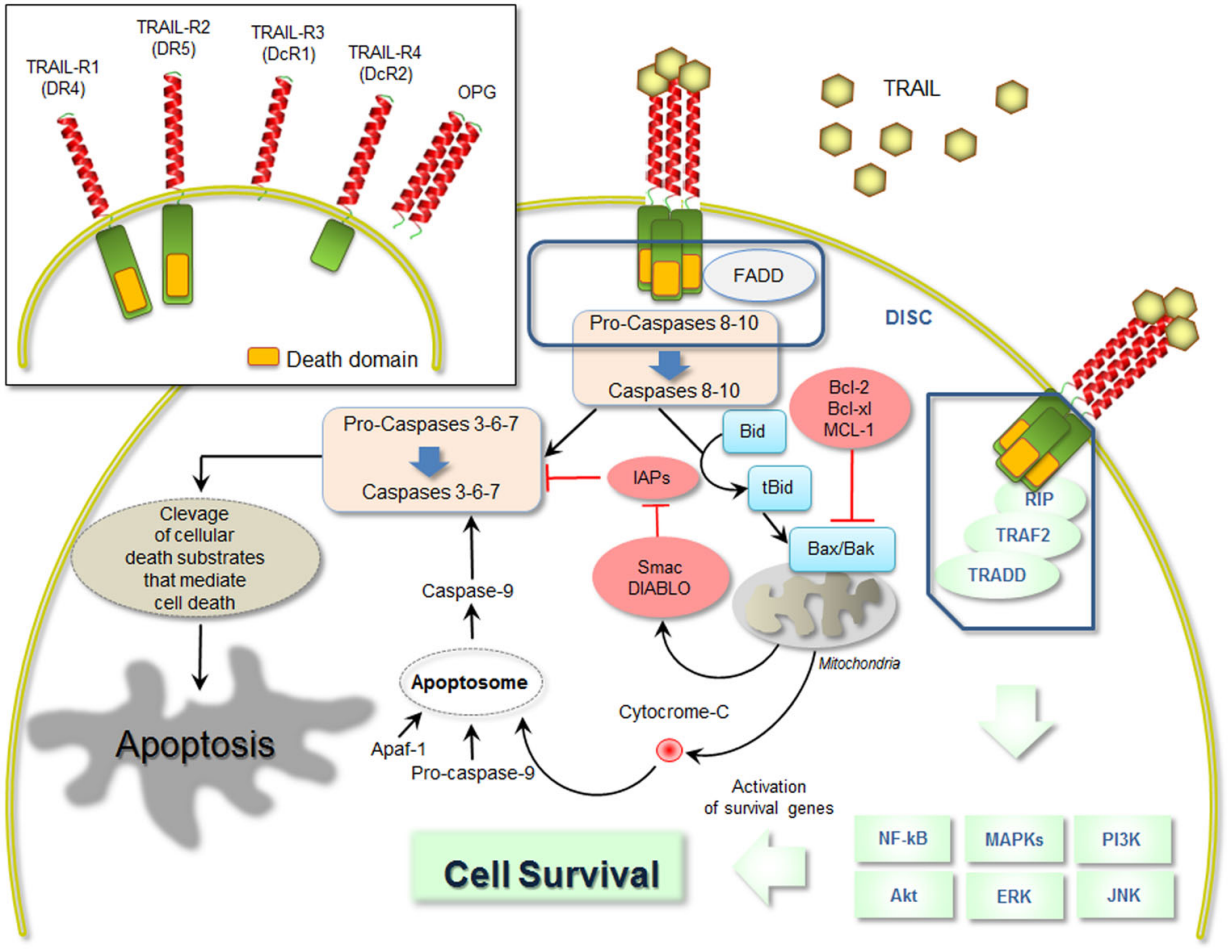
Schematic representation of TRAIL signalling pathways. The picture is a schematic representation of the main molecular mediators involved in the TRAIL-mediated apoptosis (both intrinsic and extrinsic pathways) and TRAIL-mediated cell survival, induced by the interaction between TRAIL and its receptors. On the *upper panel*, a schematic representation of the five cognate TRAIL receptors is shown. DcR1 and DcR2 are decoy receptors for TRAIL as well as the soluble osteoprotegerin

Two TRAIL-activated death pathways have been identified, an extrinsically mediated death pathway, found in cells generating enough caspase-8 (or -10) activation and apoptotic signalling to promote cell death, and a second death pathway in cells that require additional processes to lead to full apoptosis through cleavage of the pro-apoptotic protein BH3 interacting-domain (Bid) (Fig. 1). However, TRAIL can also activate other signal transduction pathways [53]. The binding of TRAIL to its cognate receptors can promote non-apoptotic, pro-survival and proliferation signals through several intracellular molecular mediators, including the transcription factor nuclear factor-κB (NF-κB), the mitogen-activated protein kinases (MAPKs), and PI3K/Akt and ERK pathways, and ultimately leading to transcription of survival genes able to regulate developmental and inflammatory processes (Fig. 1) [54, 55]. For initiation, such pathways rely on the recruitment of adaptor molecules such as TNF-receptor-associated death domain protein (TRADD), TNF-receptor-associated factor-2 (TRAF2), receptor interacting protein (RIP1), and apoptosis inhibitor proteins, which assemble into a complex that mediates intracellular signal transmission (Fig. 1).

The “cell selectivity” of TRAIL in inducing apoptosis is directed towards transformed/infected cells, leaving non-tumor cells largely unaffected, and has therefore been the focus of much scientific interest. However, increasing experimental evidence suggests that TRAIL might have several alternative roles in normal cells/tissues. Our group has already shown that TRAIL promotes the survival/proliferation of endothelial cells, suggesting that the TRAIL/TRAIL-R system plays an important role in endothelial cell physiology and the biology of the vascular system [56], and affects the survival, migration and proliferation of vascular smooth muscle cells through activation of specific intracellular pathways [57]. TRAIL can also promote pro-survival/proliferative effects and interfere with differentiation processes in several other cell types, including multipotent stem cells, osteoclasts, myeloid cells and intestinal cells [58–63], and can even, paradoxically, activate pro-survival pathways in sensitive neoplastic cells [55]. These contrasting biological effects (proliferation/survival versus apoptosis) seem to depend on the type of cells involved, and remains one of the most fascinating features of TRAIL biology.

In this regard, one hypothesis considers the different cell-related susceptibility to TRAIL as the result of different patterns of TRAIL receptors expression, specifically a different ratio between pro-apoptotic (DR) and decoy (DcR) receptors [54, 64]. The concept of “lipid rafts” has been introduced to describe plasma membrane platforms involved in mediate death receptor signals [65]. Structurally, these are dynamic pools rich in cholesterol and sphingolipids. They are able to trigger TRAIL-DISC-initiated intracellular apoptotic signals, and some reports suggest that a redistribution of TRAIL receptors and DISC composition from “non rafts” to “lipid rafts” could explain the switching between proliferative/survival and apoptotic TRAIL-mediated cell pathways in different cellular contexts [65–68]. Unsurprisingly, both the role and distribution of TRAIL receptors and the ratio between DR and DcR are still under debate and evaluation [69, 70].

The expression of TRAIL/TRAIL receptors in the brain has been analyzed by two independent groups, finding that the cytokine is virtually absent in healthy tissue, with only low levels of TRAIL ligand expression on oligodendrocytes [71, 72]. Nevertheless, both groups found similar DR4 and DR5 expression profiles, but some differences in patterns of expression of decoy receptors [71, 72]. In particular, Cannella et al. reported oligodendrocytes and neurons as the main cells expressing DcR1, and microglia the main cells expressing DcR2, while Dorr and colleagues reported that DcR1 and DcR2 were predominantly expressed by neurons and oligodendrocytes/neurons, respectively [71, 72]. This different pattern of expression of the components of the TRAIL-system in the CNS, differentially regulated in the presence of a local disease, suggest the presence of TRAIL-mediated mechanisms in the CNS. Moreover, evidence that brain tissue is susceptible to TRAIL-induced apoptosis [73] raises the hypothesis of a potential dual role of TRAIL in the CNS: on one hand leading to the activation of apoptotic pathways behind cell damage and disease progression, and on the other an overall protective/pro-survival effect, supporting the manipulation of the TRAIL system for therapeutic purposes [74].

### Perspectives of TRAIL in Alzheimer’s disease

Alzheimer’s disease (AD) is an irreversible, progressive degenerative disorder that leads to gradual cognitive impairment characterized by memory loss, reduction of intellectual abilities, changes in personality and behavior, and onset of dementia [75]. Occurring in hereditary and sporadic forms, the clinical features of AD are the result of the gradual malfunctioning and death of neurons, mainly in the cortex and hippocampus, due to accumulation of extracellular amyloid plaques and intracellular neurofibrillary tangles [75, 76]. Inflammatory processes are also involved in the pathological progression of the disease, and may even be the cause or driving force behind it [77]. In this regard, TRAIL is specifically expressed in the brains of AD patients, which display a TRAIL-specific immunoreactivity mainly localized in AD-affected regions, such as the cerebral cortex, often in the proximity of Congo-red-positive amyloid plaques [78]. Furthermore, preclinical in vitro cellular models have implicated TRAIL in the beta-amyloid protein-dependent cell death known to contribute to the neurodegenerative process and associated chronic inflammation characteristic of AD [79]. Indeed, specific blockade of the TRAIL death receptor DR5 has been shown to completely prevent amyloid beta-related neurotoxicity in two cellular models, a neuronal cell line and primary cortical neurons, suggesting a key role for DR5 in the TRAIL-induced death pathway in AD [80]. Moreover, the vascular deposition of amyloid beta, which alters cerebral blood flow and thereby contributes to cognitive impairment in AD seems to be mediated by DR4 and DR5 death receptors, via both caspase-8- and caspase-9-triggered apoptotic pathways in human cerebral microvascular endothelial cells [81].

More recently, the role of TRAIL as a mediator of amyloid-beta neurotoxicity has been explored as a potential therapeutic target for the treatment of AD in a triple transgenic mouse model of AD [82]. In their work Cantarella and colleagues have demonstrated that blocking TRAIL release and activation of apoptotic TRAIL receptors by means of a neutralizing monoclonal antibody can attenuate amyloid-beta-induced neurotoxicity [82]. In particular, treatment via intraperitoneal infusion started 6 months before the onset of the disease led to reduced amyloid-beta deposition, functional improvement, and a reduction in the inflammatory response in the brain, resulting in a significant overall improvement in cognitive parameters [82].

Evidence that there is no difference in circulating levels of TRAIL between AD patients and healthy controls seems to suggest that this effect is local and restricted to the CNS [83]. It may be that TRAIL is released by neurons in response to local stress generated by the accumulation of oligomers of amyloid-beta. Alternatively, it could be released by infiltrating immune cells or other cell types reacting to amyloid-β oligomers or increased neuronal signals [84]. Although several aspects of this approach evidently still need to be refined, this can nevertheless be considered a proof of concept that targeting amyloid pathology via manipulation of TRAIL pathways may be a new therapeutic approach for the clinical management of AD patients.

### Perspectives of TRAIL in multiple sclerosis and secondary cognitive impairment

Multiple sclerosis (MS) is a demyelinating disease of the central nervous system, and primarily the result of an auto-inflammatory reaction, although genetic predisposition and environmental factors may be implicated [85] and it still represents one of the major causes of chronic neurological disability. Even though its own etiology remains unclear, it is known, however, that the major feature of the disease is the disruption of the myelinated tracts of the CNS by auto-reactive lymphocytes [86]. This leads to cumulative and irreversible damage, as well as progressive disability [86]. In its early stages, this pathological cascade appears to be driven by a peripheral immune response targeting the CNS, followed by a later progressive phase characterized by local immune reactions within the CNS [87]. According to a recent review of the state of the art in MS, the main processes leading to neurodegeneration include microglia activation, chronic oxidative injury, accumulation of mitochondrial damage in axons, and age-related iron accumulation in the human brain [88].

Research in the field of MS takes advantage of the availability of murine models of the disease, including experimental autoimmune encephalomyelitis (EAE), providing insights into the biological processes and development/progression of MS [89]. Although no single model can replicate the complexity of MS and all its pathogenic pathways [89], preclinical evidences show that blockade of the TRAIL-pathway in mice by various approaches exacerbated EAE, increasing both disease score and the degree of inflammation in the CNS, while treatment with recombinant soluble TRAIL delayed disease onset and reduced the severity of EAE [90, 91]. It appears that TRAIL-mediated effects were not related to apoptosis induction in inflammatory cells, but rather to the prevention of autoreactive T cell activation [90–92]. In addition to its influence on T cell growth/functionality, there is evidence that TRAIL is involved in triggering and promoting CD4^+^CD25^+^ regulatory T cells [93]. Nevertheless, a TRAIL-mediated contribution to cell damage in the CNS in the presence of neuroinflammation has also been reported [94], confirming that this molecule has a dual role, provoking different outcomes when peripheral or central tissues are involved and inducing contrasting effects at different stages in the course of the disease. It is evident that these two “sides” of TRAIL, anti-inflammatory and pro-apoptotic, could be targeted differently for the same therapeutic purpose in EAE/MS, and, indeed, the therapeutic potential of targeting the TRAIL system to manage EAE has recently been explored using different approaches. In one study, a fusion protein between the extracellular domain of fibroblast growth-factor-inducible 14, formally the cell surface receptor for the TNF family member inducer of apoptosis TWEAK, and the extracellular domain of TRAIL was designed as an anti-inflammatory agent [95]. Injected daily at different time points after disease induction in mice, the fusion protein brought about clinical improvement correlated with: (1) decreasing lymphocyte infiltrate in the CNS; (2) decreased Th1/Th17 responses, and (3) increased number of regulatory T cells [95]. Similarly, prior to the onset of EAE, intrathecal delivery of a plasmid DNA coding for a fusion protein between TRAIL and OX40, a member of the TNF receptor super family predominantly expressed on activated T cells, reduced disease severity and inflammatory cell infiltrates [96].

As higher TRAIL levels have been reported in established MS lesions [71, 72], and affected brains show a different pattern of death receptor expression than healthy controls, the TRAIL system could play an important role in the pathological process of MS [71, 72]. In the light of the body of evidence coming from preclinical EAE model, attention has turned to TRAIL as a potential clinical biomarker in MS, focusing also on the possible link between gene polymorphisms of TRAIL system components and the clinical course of the disease. Although a recent study reported no difference in serum TRAIL levels between MS patients and healthy controls, comparative analysis of patients reporting different clinical forms and activity phases of the disease showed that TRAIL is significantly reduced during relapse in relapsing-remitting MS patients [97]. In the same context, other authors have reported lower serum levels of soluble TRAIL protein in MS patients with respect to healthy controls but they found no differences in the expression ratio of the TRAIL mRNA gene expression ratio [98].

These discrepancies suggest the need for larger cohort of patients and appropriate functional studies, in particular to investigate whether the TRAIL levels observed in MS patients are associated with enhanced survival of pathogenic T lymphocytes and other inflammatory markers. Moreover, since TRAIL has been suggested to be involved in Interferon beta (IFN-β) activity, the assessment of its expression has been proposed as potential prognostic marker of treatment response to IFN-β in MS patients [99, 100]. Other lines of evidence suggest that genetic variants of the TRAIL and TRAIL-receptor genes may be associated with MS susceptibility as reported by studies considering different single-nucleotide polymorphisms and splice variants in different cohorts of patients [101–103]. However, larger sample sizes are required to confirm the preliminary results indicating that splice variants of the TRAIL system could have a potential as predictors of IFN-β treatment in MS patients [104].

The role of TRAIL has also been evaluated in other cognitive impairment conditions, often secondary manifestations of specific primary diseases such as viral infection, or the result of distress related to depression or drug use. For instance, despite considerable advances in treatment, neurocognitive impairment still occurs in HIV-infected subjects, whether asymptomatic, mild neurocognitive disorder or HIV-associated dementia (HAD) [105]. This may be due to the virus infecting the perivascular macrophages and microglia in the brain and the release of viral proteins that directly kill neurons [105], or inflammation and neuron loss may be ascribable to inflammatory mediators released from the macrophages and microglia activated in response to HIV infection [105]. In the context of HAD, TRAIL has been shown to actively participate in neuron loss [106] by inducing apoptosis in HIV-1-infected macrophages and cultured neurons [106]. TRAIL levels are increased in human monocyte-derived macrophages after HIV-1 infection and it is known to be mediated by IRF-1, IRF-7, Type-I IFNs, and STAT-1 [107].

In psychiatric disorders, which are associated with neuroendocrine changes, a recent study has evaluated the pattern of expression of circulating cytokines in the search for potential biomarkers [108]. The results show that 13 cytokines, including TRAIL-R4, are overexpressed in individuals with major depressive disorder reporting childhood trauma, suggesting a relationship between depression and cytokine alterations [108]. Moreover, in line with the evidence that drug abuse affects the inflammatory system, another recent study has analyzed the plasma levels of soluble receptors/ligands in cocaine-dependent subjects experiencing or not early life stress in comparison with control subjects [109]. TRAIL levels were higher in cocaine-dependent subjects experiencing early life stress, which, although failing to reach statistical significance, does indicate a possible link with circulating levels of pro-inflammatory mediators [109].

### Perspectives of TRAIL in ischemic stroke

Ischemic stroke results in a cascade of events involving neuronal death, alteration of the white matter pathophysiology, and local inflammation. The resulting clinical picture is so complex that, while trials testing a preventive approach have gained major successes in recent years, the search for effective acute stroke treatments has encountered many failures. This suggests that successful translation of biological/pathophysiological knowledge into effective clinical approaches will definitely require an integrative approach [110]. In particular, the cerebral inflammation, the biological significance of its resolution, and the repair of neuronal damage, have been recently reviewed focusing in particular on the role of the so-called DAMPs, i.e., molecules released from damaged cells that play a key role in triggering local inflammation [111]. During ischemic stroke, activation of the microglia is followed by local recruitment of leukocytes to the brain lesion, thereby contributing to the tissue injury pathogenesis [112–114]. In this context, TRAIL seems to play a role in inducing apoptosis, as evidenced in preclinical models [115, 116] that highlight the involvement of TRAIL in neuronal apoptosis in vivo after ischemia. This apparently occurs through a c-Jun-mediated signalling pathway that was completely prevented by immunosuppressant treatment [115].

In a murine model of global cerebral ischemia, expression and upregulation of both TRAIL and its receptor DR5 have been shown after transient ischemia–reperfusion [117]. Expression of TRAIL was more selective to astrocytes and activated microglia/macrophages, while the DR5 receptor was more predominantly expressed in neurons [117]. In the same model, functionally inhibiting TRAIL by blocking its interaction with DR5 led to reduced apoptosis and a neuroprotective effect overall [117]. Other recent studies have confirmed the overexpression of TRAIL and TRAIL receptors in hypoxic brain [118, 119], and the local expression of decoy receptors for TRAIL has been shown to confer neuronal protection against lethal ischemia after ischemic preconditioning [120]. In particular, Cantarella and colleagues have shown that TRAIL is upregulated in focal ischemia, while it appears to be downregulated in sub-lethal brain ischemia and brains subjected to preconditioning stimulus [119]. In this model, neuroprotection elicited by ischemic preconditioning was found to occur through both upregulation of TRAIL decoy receptors and downregulation of its death receptors, as well as TRAIL itself, which indicated that TRAIL inhibition may significantly limit tissue damage and promote functional recovery [119].

Recent clinical trials have assessed TRAIL as a potential biomarker for acute stroke and stroke subtypes. However, a study performed in plasma samples of patients with recent lacunar infarcts (silent brain infarcts) revealed no significant difference in TRAIL levels with respect to healthy controls [121]. Nonetheless, in a recently published study performed in a cohort of patients with acute ischemic stroke, significant negative correlations between serum TRAIL levels and both clinical score and stroke volume have been reported, with no difference between the various stroke subtypes analysed, in line with findings reported in other cardiovascular diseases [122].

## Concluding remarks

**Fig. 2:**
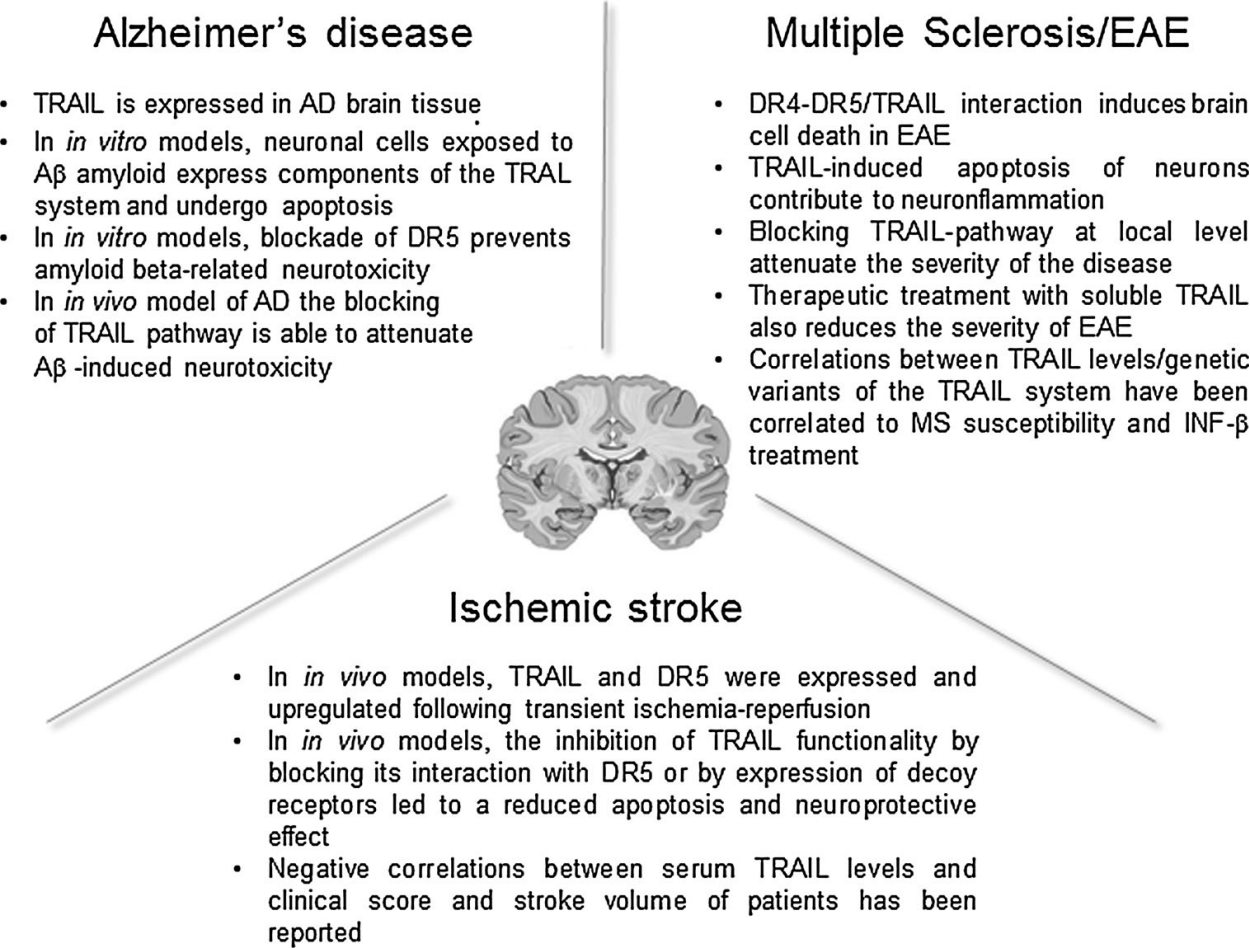
Snapshot of the main preclinical evidences of the involvement of TRAIL in neuroinflammatory diseases. The picture shows some of the most interesting features coming from the preclinical models/evaluations about the role of TRAIL in the context of Alzheimer’s disease, multiple sclerosis and ischemic stroke in view of a future clinical development of TRAIL-based therapeutic strategies

In line with findings obtained in other inflammatory conditions, the overall message from the recently published results is that TRAIL also plays a dual role in inflammatory disorders of the CNS, likely as the result of different involvement/effects of TRAIL pathway in local and peripheral inflammatory processes. In terms of future clinical perspectives, therefore, TRAIL appears to be an attractive therapeutic target, and TRAIL-based approaches interfering with TRAIL pathways have already demonstrated therapeutic potential, particularly in Alzheimer’s disease and EAE/MS (Fig. 2). Moreover, preliminary evidence seems to suggest a potential role of circulating TRAIL as a biomarker for CNS-related disorders such as neurocognitive impairment and depression. Nevertheless, further investigation is required in order to: (1) clarify the local molecular insights into the mechanisms of action of TRAIL; (2) evaluate possible TRAIL-based treatment strategies, taking opportunely into account the different and complex immunological features of the specific neurological disorders; and (3) develop appropriate delivery systems for both systemic and local approaches based on the specific molecular/clinical features of the diseases in question.
